# A *TMEM63A* Nonsense Heterozygous Variant Linked to Infantile Transient Hypomyelinating Leukodystrophy Type 19?

**DOI:** 10.3390/genes15050525

**Published:** 2024-04-23

**Authors:** Dimitra Siori, Dimitrios Vlachakis, Periklis Makrythanasis, Joanne Traeger-Synodinos, Danai Veltra, Afrodite Kampouraki, George P. Chrousos

**Affiliations:** 1 University Research Institute of Maternal and Child Health and Precision Medicine, School of Medicine, National Kapodistrian University of Athens, 115 27 Athens, Greece; demisior@gmail.com (D.S.); dimvl@aua.gr (D.V.); 2 Clinical and Translational Research Endocrine Unit, School of Medicine, National Kapodistrian University of Athens, 115 28 Athens, Greece; 3 Laboratory of Genetics, Department of Biotechnology, School of Applied Biology and Biotechnology, Agricultural University of Athens, 75 Iera Odos, 11855 Athens, Greece; 4 School of Informatics, Faculty of Natural & Mathematical Sciences, King’s College London, Bush House, Strand, London WC2R 2LS, UK; 5 Laboratory of Medical Genetics, School of Medicine, National Kapodistrian University of Athens, 115 27 Athens, Greece; 6 Department of Genetic Medicine and Development, Medical School, University of Geneva, 1211 Geneva, Switzerland; 7 Biomedical Research Foundation of the Academy of Athens, 115 27 Athens, Greece

**Keywords:** leukodystrophy, myelin, oligodendrocyte, hypomyelination, remyelination, infantile transient hypomyelinating leukodystrophy type 19, motor delay, developmental delay, TMEM63A variant

## Abstract

Infantile onset transient hypomyelination (IOTH) is a rare form of leukodystrophy that is associated with transient motor impairment and delayed central nervous system myelination. Here, we report a case of a new mutation in the transmembrane protein 63A (*TMEM63A*) gene identified using Whole-Exome Sequencing (WES) in an 8.5-year-old boy with clinical symptoms similar to IOTH. The patient exhibited a mild developmental delay, including hypotonia and delayed motor milestones, as well as some notable phenotypic characteristics, such as macrocephaly and macrosomia. Despite the absence of early neuroimaging, genetic testing revealed a paternally inherited variant in *TMEM63A* (NM_14698.3:c.220A>T;p:(Arg74*)), potentially linked to infantile transient hypomyelinating leukodystrophy type 19. Our findings in this study and the patient’s favorable clinical course underscore the potential for successful myelination even with delayed initiation and may contribute to a better understanding of the genotype–phenotype correlation in IOTH, emphasizing the importance of genetic analysis in unresolved developmental delay cases and providing critical insights for accurate diagnosis, prognosis and potential therapeutic strategies in rare leukodystrophies.

## 1. Introduction

Leukodystrophies are rare neurological disorders that are primarily characterized by deficiencies in the formation of myelin (CNS), which plays a crucial role in the efficient transmission of electrical signals between neurons [1]. The term “leukodystrophy” derives from the Greek language, meaning “the abnormal growth of white matter”. Myelin production starts during the third trimester of pregnancy and rapidly accelerates postnatally and during early childhood. By the age of 2 years, most of the myelination process is complete. However, it continues at a slower rate into adulthood [2,3]. The incidence of leukodystrophies ranges from 1/8000 to 1/80,000 [4], and they are often associated with regression or the loss of developmental abilities, such as speech and motor milestones, as well as with cognitive defects. Although most of them present during childhood, some are adult-onset, which may reflect diverse etiologies and disease mechanisms and is an ongoing topic of research.

Leukodystrophies have various genetic causes with different inheritance patterns, including autosomal recessive, autosomal dominant and X-linked recessive traits. Depending on the affected gene studied, the therapeutic approaches include replacement therapies and gene suppression [5,6]. The mutations associated with leukodystrophies usually affect the pathways involved in the production or breakdown of myelin lipids and lysosomal enzymes that participate in lipid metabolism (Fabry disease, fucosidosis and Krabbe disease) [7]. Peroxisomal proteins, which contribute to the β-oxidation of fatty acids and the synthesis of plasmalogens, are also implicated in multiple leukodystrophies, including X-linked adrenoleukodystrophy [8]. The other molecular mechanisms associated with leukodystrophies include defects in the mitochondrial proteins (cerebrotendinous xanthomatosis); cytoskeletal proteins (autosomal dominant leukodystrophy with autonomic disease); transcription (4H leukodystrophy) and translation (tRNA synthetase-related leukodystrophies); myelin structural proteins (CNP-related hypomyelinating leukodystrophy and Pelizaeus–Merzbacher Disease); cell junction; and other transmembrane proteins [9,10,11,12,13,14,15,16,17]. While genetic causes play a significant role in white matter development and disease, a neonatal white matter injury or congenital heart disease can also adversely affect neurodevelopment and lead to long-lasting effects. Maternal infection and perinatal inflammatory insults are also associated with a reduction in the expression of oligodendrocyte differentiation and cerebral palsy [18,19,20,21].

Infantile onset transient hypomyelination (IOTH) or infantile transient hypomyelinating leukodystrophy is a rare genetic neurological disorder that affects myelin formation in the CNS, causing temporary motor impediment [22,23,24]. It presents in infants with hypotonia and a delay in developmental milestones, such as sitting, crawling and walking. The other symptoms include nystagmus, ataxia, dysmetria, an intention tremor, hearing deficiencies, ocular abnormalities and paroxysmal events with spinal cord involvement in rare cases [22,23,24]. IOTH is generally transient and self-limiting, with the symptoms resolving as myelination catches up to normal developmental timelines. IOTH is due to pathogenic variants in several genes, most frequently *POLR3A* and *POLR3B*, which encode RNA polymerase III, a key enzyme for the normal formation of the transcription machinery, and therefore the development and maintenance of myelin in the CNS [25].

The diagnosis of IOTH is based on neuroimaging studies, particularly the magnetic resonance imaging (MRI) of the brain and spinal cord, which shows a characteristic pattern of delayed myelination in the white matter of the brain [22,23,24]. The differential diagnosis of IOTH includes other leukodystrophies or genetic disorders affecting myelin that have similar symptoms and imaging patterns. The clinical and neuroradiological presentation of IOTH is similar to Pelizaeus–Merzbacher Disease (PMD), which is caused by changes in the gene that encodes for proteolipid protein 1, a structural myelin protein. However, PMD patients have a less-favorable clinical course and developmental progress compared to IOTH patients [26]. There is no specific cure for IOTH, and supportive care is the primary management strategy, as the disease is transient, and its prognosis is relatively favorable [22,23,24].

Here, we present a patient with suspected IOTH type 19 caused by a novel nonsense variant in the gene encoding transmembrane protein 63A (*TMEM63A*). While the *OSCA*/*TMEM63* family had not been associated with any human disease, only five years ago, in 2019, Yan et al. pointed out a connection between the mutations in *TMEM63A* and infantile onset transient hypomyelination [22]. In addition, we briefly review other published data on the known *TMEM63A* variants.

## 2. Materials and Methods

### 2.1. Clinical Data Collection

This study involved a Greek family that included a boy with a history of developmental delay and his relatives. Clinical examination of the patient and his parents was followed by genetic counseling. After the parents signed an informed consent form, blood samples were obtained for genetic testing. The study was approved (RPURI9002) by the Bioethics Committee of the University Research Institute of Maternal and Child Health and Precision Medicine at the School of Medicine of the National Kapodistrian University of Athens. All the images were published after paternal consent was obtained.

### 2.2. DNA Sequence Analysis

Genomic DNA was isolated from the white blood cells of the patient and his parents using a Nucleospin^®^ Blood Quickpure kit (Macherey Nagel GmbH, Düren, Germany) following the guidelines of the manufacturer. Subsequently, Whole-Exome Sequencing (WES) was performed on the patient’s DNA sample for the identification of the mutation using NextSeq-500 (Illumina, San Diego, CA, USA). Variation analysis was performed using the VarSome Clinical platform and varAFT 2.14 (http://varaft.eu, accessed on 8 May 2022) that uses genotype–phenotype correlation predictions from several genetic databases. For confirmation, the targeted DNA sequencing of the *TMEM63A* gene region containing the found mutation was carried out for the patient and his parents using an automated capillary sequencer ABI 3730 XL Analyzer (Applied Biosystems, Waltham, MA, USA).

## 3. Results

### 3.1. Case Report

**Figure 1:**
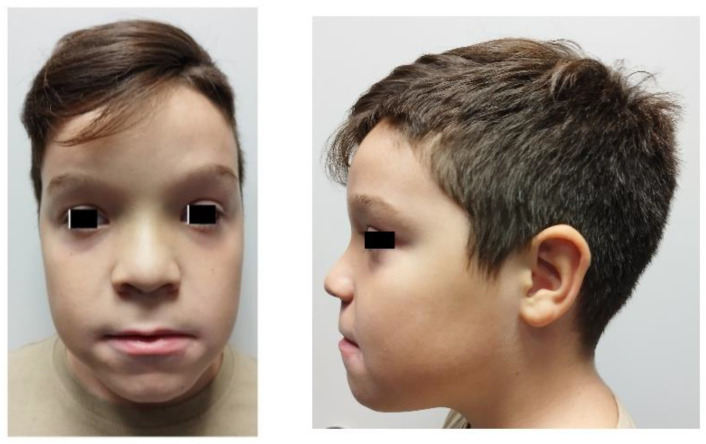
The phenotype of the patient.

An 8.5-year-old boy was clinically evaluated at the Clinical and Translational Research Endocrine Unit, School of Medicine, of the National Kapodistrian University of Athens. The boy was born by normal delivery at 35 weeks gestational age due to placental abruption. His birth weight was 2.7 kg, and no post-partum complications occurred. His parents were Greek with self-reported good health and unrelated, but originated from the same agricultural region. The boy presented with macrosomia, macrocephaly, a large forehead, low-set ears and a depressed nasal bridge as well as hypotonia during infancy and delayed motor development (Figure 1). His cognitive condition was normal, but his speech speed was mildly slow. No endocrine disorders, including hypo- or hyperthyroidism, were detected. 

**Figure 2:**
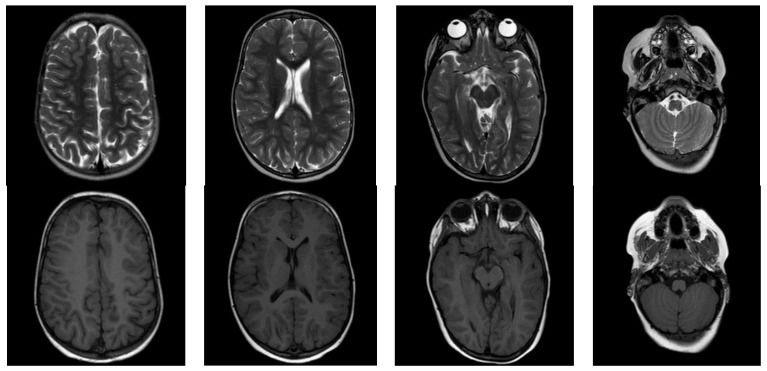
Serial MRIs (axial T2- and T1-weighted images) of the individual at 3 years of age show normal myelination. No abnormalities are depicted here.

The boy had a history of developmental delay. The following milestones were was achieved: head control at age 6 months, sitting without a support at 10 months, walking without a support at 18 months, and climbing the stairs at 36 months. It is important to mention that physiotherapy was initiated at the age of 6 months after a neurologist’s recommendation and is still ongoing. Communication milestones were achieved without significant deviation. He started to make meaningful sounds at age 9 months and said two or more words in a sentence at age 17 months. He was able to speak long sentences with mildly slow pronunciation. His cognitive performance was spared, without any noteworthy observations. At the age of 3 years, genetic testing for Prader–Willi syndrome had negative results, while brain MRI did not reveal myelination abnormalities or other types of disorders (Figure 2). It should be noted that, unfortunately, MRI was performed relatively late for this case. 

### 3.2. Molecular Genetic Analysis

**Figure 3:**
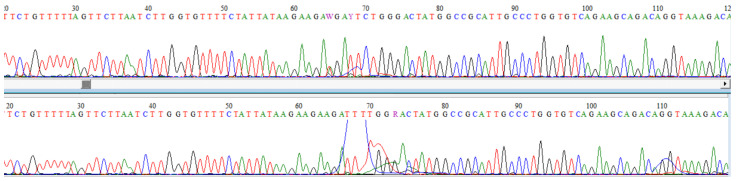
Sanger sequencing of the mutation (c.220A>T;p:(Arg74*)) in *TMEM63A.*.

After genetic counseling and signed informed consent was given, we obtained blood samples from the patient and his parents for genomic DNA isolation and genetic testing. The WES analysis of the patient’s DNA detected a novel heterozygous nonsense variant in the gene encoding transmembrane protein 63A *TMEM63A* (NM_014698.3:c.220A>T;p:(Arg74*)), potentially underlying the diagnosis of infantile transient hypomyelinating leukodystrophy type 19 (OMIM 618688). Targeted Sanger DNA sequencing of the *TMEM63A* gene region (Figure 3) was carried out for him and his parents, confirming the finding from the patient’s DNA and revealing that the mutation was paternally inherited. Further testing showed that the variant was also present in the paternal grandmother.

### 3.3. Follow-Up

Records from the father’s and grandmother’s childhoods were unavailable to determine possible clinical similarities in the infantile period. In light of the genetic testing results, genetic counseling was offered to the family. The parents reported relief that the previously unknown case of their child’s condition was finally resolved. In a follow-up of our patient, who is now a 10-year-old boy, we have observed that he is able to walk, run, and climb the stairs without difficulties, and his communication and cognitive skills are concordant with his age at evaluation. A recent physical examination demonstrated no pathological semeiology, besides the noteworthy facial phenotype described above. Finally, the results of additional routine investigations were normal, and no endocrine or metabolic disorders were revealed, nor was the child’s growth rate affected. 

## 4. Discussion

**Figure 4:**
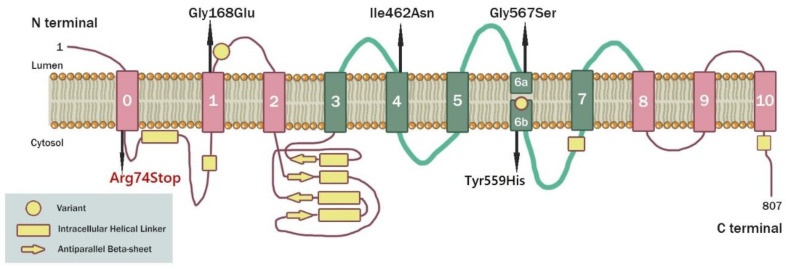
A schematic representation of a TMEM63A protein and the location of the identified variants causing infantile transient hypomyelinating leukodystrophy type 19. Modified version of the representation by Yan et al., 2019 [22].

We present a patient with possible infantile transient hypomyelinating leukodystrophy type 19 caused by a novel nonsense variant in the gene encoding transmembrane protein 63A (*TMEM63A*) (Figure 4). TMEM63A is a mechanically activated ion channel that belongs to the osmosensitive calcium-permeable OSCA/TMEM63 family of channels, which is conserved across eukaryotic species and contains two more members, TMEM63B and TMEM63C [27,28,29]. TMEM63A is highly expressed in oligodendrocytes both in humans and in mice [22,27,28,29]. Mice lacking the TMEM63A gene exhibit abnormalities in their gait, as noted in the International Mouse Phenotyping Consortium (IMPC) database [22,29]. TMEM63A is the first member of the OSCA/TMEM63 family to be associated with a human disease [22]. It is possible that the transient nature of this condition is due to the developmental and tissue-specific expression of TMEM63A’s homologs, TMEM63B and TMEM63C, which compensate, at some level, for the loss of TMEM63A’s activity [29]. The formation of an ion channel properly activated by hyperosmolarity requires the expression of all three TMEM63 proteins, and this fact may be crucial in certain developmental processes [30]. However, research on TMEM63A’s role in disease is still ongoing, and since it is known to be expressed in various tissues throughout the body, its exact function and implications in human health may not have been fully elucidated yet [27,28,29].

The present study involves a novel heterozygous nonsense variant in the *TMEM63A* gene (NM_014698.3:c.220A>T:p:(Arg74*)), which is suspected to cause autosomal dominant infantile transient hypomyelinating leukodystrophy type 19 (IOTH19, OMIM 618688). All five previously reported patients with IOTH19 due to mutations in *TMEM63A* carried heterozygous missense variants, including four de novo and one inherited one. Specifically, in 2019 Yan et al. was the first to identify heterozygous missense mutations in *TMEM63A* in four unrelated patients, three of whom had a de novo variant, and one was paternally inherited [22], and in 2021, Tonduti et al. reported a de novo heterozygous missense mutation in *TMEM63A* in a 15-month-old girl (Figure 3) [23]. Our patient is the first one with an inherited nonsense variant in *TMEM63A*. It is yet to be ascertained whether this deviation is crucial and responsible for the clinical differentiation of our case. Moreover, the presented patient had birth complications, specifically placental abruption, while the other cases reported depicted a range of diverse birth scenarios, including high-risk pregnancies due to gestational diabetes and completely uneventful pregnancies and births.

The diagnosis of infantile transient hypomyelinating leukodystrophy type 19 is characterized by a temporarily impaired motor ability and hypomyelination on MRI, which typically improves after the first two years of life. However, in the patient presented here, no MRI was performed before the age of 3 years old, and hence no image proving hypomyelination was given, providing our study with a significant limitation to confirm the delay in myelin formation. Therefore, the effect of the variant on myelin formation can only be suspected from the clinical observations and not from the radiological findings. 

Our patient’s clinical picture was similar to most IOTH cases previously described [22,23,24]. Nevertheless, he followed a milder course with a favorable outcome, without epileptic events or intellectual disability. Additionally, our patient displayed a distinct phenotype, which includes macrosomia, macrocephaly, a large forehead, low-set ears and a depressed nasal bridge. These features could serve as essential diagnostic clues. However, it is still unknown and further investigation is necessary to determine if these characteristics are common among individuals with this condition, or if they are only present in our case. While the clinical trajectory of other documented cases varies, with some displaying residual permanent pathology, such as optic nerve atrophy, this case exhibited a favorable clinical evolution, despite having this damaging genetic variant. This emphasizes the heterogeneity of this type of disorder, still provides important prognostic information for affected families, and highlights the potential for successful myelination even when it is initiated later than usual, offering hope for therapeutic trials in hypomyelinating disorders.

Due to the difficulty of diagnosing based solely on the clinical manifestations, WES was used to provide a genetic diagnosis of infantile transient hypomyelinating leukodystrophy type 19. This underscores the significance of genetic analysis methods in unexplained developmental delay cases.

There is no specific cure for IOTH, and supportive care is the primary management strategy, which includes physical, occupational and speech therapies to address developmental delays and motor difficulties. Regular follow-ups and monitoring are necessary to track the progress and make adjustments to the care plans. The prognosis for IOTH varies, but it can be relatively favorable due to the transient nature of the disease. While some children experience a significant improvement in their symptoms and developmental progress, others may have ongoing neurological challenges. The long-term outlook often depends on the specific genetic mutations involved and the extent of myelin recovery. However, in most cases, like the one presented here, children improve over time and have a relatively normal quality of life.

## 5. Conclusions

Our study has uncovered a novel nonsense variant potentially linked with infantile transient hypomyelinating leukodystrophy type 19, representing a valuable addition to the growing body of knowledge on the complex genetic underpinnings of this condition. Also, by presenting detailed clinical characteristics, we may improve the genotype–phenotype correlation in the literature for rare and novel variants, and therefore make a critical step toward improved the diagnostic accuracy, prognosis and potential therapeutic strategies, ultimately benefiting the affected children and their families. Although we were unable to evaluate the first diagnostic and later follow-up neuroimaging studies in our case, WES combined with our clinical suspicion led to a potential diagnosis. This highlights the importance of Next-Generation Sequencing in reducing the number of unsolved cases and associating clinical manifestations with molecular pathways, especially in conditions with significant heterogeneity.

## Data Availability

The original contributions presented in this study are included in the article. Further inquiries can be directed to the corresponding authors.
